# Reconstructing a Flavodoxin Oxidoreductase with Early Amino Acids

**DOI:** 10.3390/ijms140612843

**Published:** 2013-06-19

**Authors:** Ming-Feng Lu, Hong-Fang Ji, Ting-Xuan Li, Shou-Kai Kang, Yue-Jie Zhang, Jue-Fei Zheng, Tian Tian, Xi-Shuai Jia, Xing-Ming Lin, Hong-Yu Zhang

**Affiliations:** 1 School of Life Sciences, Shandong Normal University, Jinan 250014, China; 2 School of Life Sciences, Shandong University of Technology, Zibo 255091, China; E-Mails: jhf@sdut.edu.cn (H.-F.J.); zhangyuejie@sdut.edu.cn (Y.-J.Z.); 3 National Key Laboratory of Crop Genetic Improvement, College of Life Science and Technology, Huazhong Agricultural University, Wuhan 430070, China; E-Mails: ltx19870901@163.com (T.-X.L.); kangpp006@163.com (S.-K.K.); zhengjuefei25@163.com (J.-F.Z.); ttlym1989@gmail.com (T.T.); xishjia@163.com (X.-S.J.); xingminglink@gmail.com (X.-M.L.); zhy630@mail.hzau.edu.cn (H.-Y.Z.); 4 Center for Bioinformatics, Huazhong Agricultural University, Wuhan 430070, China

**Keywords:** origin of life, primitive redox protein, cofactor, early amino acid

## Abstract

Primitive proteins are proposed to have utilized organic cofactors more frequently than transition metals in redox reactions. Thus, an experimental validation on whether a protein constituted solely by early amino acids and an organic cofactor can perform electron transfer activity is an urgent challenge. In this paper, by substituting “late amino acids (C, F, M, T, W, and Y)” with “early amino acids (A, L, and V)” in a flavodoxin, we constructed a flavodoxin mutant and evaluated its characteristic properties. The major results showed that: (1) The flavodoxin mutant has structural characteristics similar to wild-type protein; (2) Although the semiquinone and hydroquinone flavodoxin mutants possess lower stability than the corresponding form of wild-type flavodoxin, the redox potential of double electron reduction *E*_m,7_ (fld) reached −360 mV, indicating that the flavodoxin mutant constituted solely by early amino acids can exert effective electron transfer activity.

## 1. Introduction

The problem of origin of life remains one of the central and most intractable problems of modern biology. Oxidoreductases or redox enzymes are enzymes that catalyze the transfer of electrons from reductants (electron donors) to oxidants (electron acceptors), which are not only vital to biological energy production but also critical to many signaling processes governing gene regulation and expression. To perform their catalytic activities, oxidoreductases often require organic cofactors (e.g., flavine adenine dinucleotide (FAD), flavine mononucleotide (FMN), nicotinamide adenine dinucleotide (NAD), nicotinamide adenine-dinucleotide phosphate (NADP), or transition metal ions (e.g., copper, iron, manganese, nickel, or cobalt) for catalysis. Given that both organic cofactors and transition metal ions are available in the pre-protein world, an interesting question arises: Which kinds of cofactors were more involved in the redox processes of primitive oxidoreductases? By analyzing the relative abundances of amino acids that constituted the binding sites of two kinds of cofactors in the primordial world and according to the evolutionary theory of genetic code, we previously found that the metallic redox proteins depended largely on late amino acids (e.g., His, Cys, and Met) to bind cofactors, whereas the nonmetallic redox proteins mainly relied on early amino acids (e.g., Gly, Ala, and Thr) to fasten redox cofactors [1]. Thus, primitive proteins are hypothesized to have utilized organic cofactors more frequently than transition metals in redox reactions [1]. However, whether a protein constituted solely by early amino acids and an organic cofactor can exert electron transfer activity has not yet been tested experimentally.

To support our hypothesis, we adopted a top-down approach in the present study to reconstruct and synthesize a redox protein constituted solely by early amino acids. The structural and redox properties of the redox protein were also tested experimentally. Furthermore, substantial progress was achieved on the issue of using specific subsets of amino acids to produce some novel function in the past few years [2–4]. However, no experiment was performed regarding the primitive redox proteins using early amino acids. Thus, the present study will not only deepen our understanding on primitive oxidoreductases but also provide several useful hints on novel redox protein design.

## 2. Results and Discussion

### 2.1. Reconstruction of the Flavodoxin Mutant

The available nuclear magnetic resonance (NMR) structure of *Megasphaera elsdenii* flavodoxin in complexes with reduced FMN (Protein Data Bank, PDB code: 2FZ5) was used as the initial structure [5]. Six amino acids (*i.e*., His, Cys, Phe, Tyr, Met, and Trp) cannot be synthesized in Miller’s spark discharge experiments and are usually recognized as late amino acids [6,7]. Thus, we substituted these late amino acids with early ones on the basis of recognized criteria. Considering that the amino acid composition of *M. elsdenii* flavodoxin does not include His, 16 positions were mutated to rebuild the design structure using the Biopolymer module of the InsightII software (Table 1). After a ~500-ps timescale equilibrating molecular dynamics (MD) simulation, the mutant retained a 3D-structure of wild-type flavodoxins, suggesting that the mutant protein may be expressed with some stability.

### 2.2. Expression and Purification of the Flavodoxin Mutant

**Figure 1:**
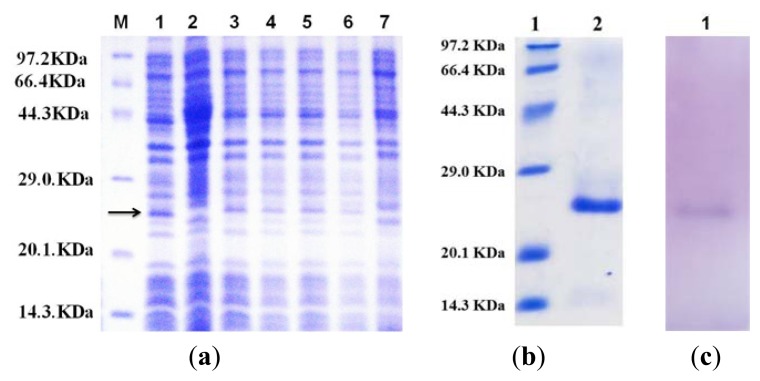
Expression and purification of the recombinant flavodoxin. (**a**) 15% SDS-PAGE analysis of expression and solubility for pET28-bF. M protein molecular mass marker; lane 1: insoluble pellet induced for 12 h by 1% isopropyl-d-thiogalactopyranoside (IPTG); lane 2: insoluble pellet of control; lane 3 to lane 6: supernatant induced for 12, 10, 8, and 6 h by 1% IPTG, respectively; lane 7: supernatant of control; (**b**) 15% SDS-PAGE analysis of the purification of the recombinant flavodoxin; (**c**) Result of Western blot. Western blotting with his-tag primary antibody (Beyotime, Haimen, China) at a 1:500 dilution and goat anti-mouse secondary antibody (Beyotime) conjugated with horseradish peroxidase (HRPO) at a 1:1000 dilution and developed with substrate solution (3′3′-diaminobenzidine tetrahydrochloride peroxidase, DAB).

The flavodoxin mutant was accumulated both in soluble and insoluble fractions of *Escherichia coli* (Figure 1a). The relative molecular mass of the recombinant protein was approximately 24 kDa, which is larger than the theoretical value (18.26 kDa). The difference should mainly arise from the effect of 6× His tag. The purified recombinant protein gave a single band after sodium dodecyl sulfate polyacrylamide gel electrophoresis (SDS-PAGE) analysis (Figure 1b), and Western blot further verified that the protein purified using affinity chromatography is the target protein containing 6× His tag (Figure 1c).

### 2.3. Spectral Assay

**Figure 2:**
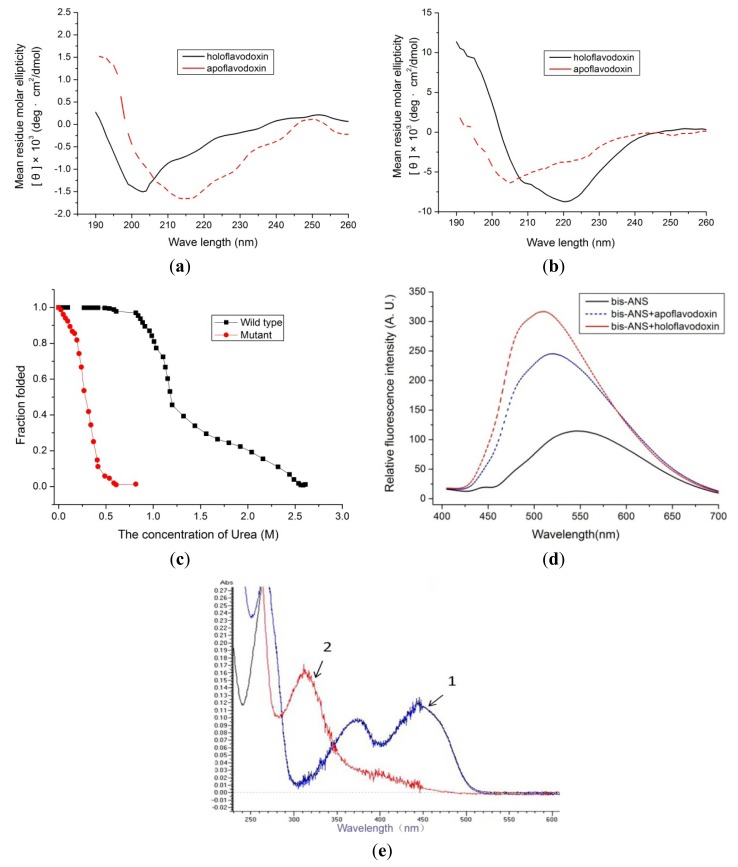
Spectral assay of the recombinant flavodoxin. (**a**) Far-UV circular dichroism spectra of the mutant apoflavodoxin and holoflavodoxin; (**b**) Far-UV circular dichroism spectra of the wild-type apoflavodoxin and holoflavodoxin; (**c**) Urea denaturation curve of the 10 μM wild-type and mutant holoflavodoxins in 5 mM morpholinopropanesulfonic acid (MOPS) buffer, pH 7.0; (**d**) Fluorescence spectra of 10 μM *bis*-ANS in the absence and presence of 10 μM mutant apoflavodoxin or holoflavodoxin; (**e**) Ultraviolet-visible spectra of reduced flavodoxin by anaerobic titration with sodium dithionite. **1**. Spectra of fully oxidized holoflavodoxin; **2**. Spectra of fully reduced flavodoxin.

The far-ultraviolet (UV) circular dichroism (CD) spectra of wild-type and mutant apoflavodoxin and holoflavodoxin are shown in Figure 2a,b. Although the CD spectra of the mutant apoflavodoxin is similar to that of the wild type, a difference between wild-type and mutant holoflavodoxin was observed, suggesting that the conformation transition of mutant and wild-type protein induced by FMN binding is different. The far-UV CD spectra show that a certain difference exists between apoflavodoxin and holoflavodoxin, indicating that the conformation rearrangement is established by the cofactor FMN in holoflavodoxin. To investigate the stability of the secondary structure of wild-type and mutant holoflavodoxin to urea-induced denaturation, far-UV CD spectra were recorded for both wild-type and mutant holoflavodoxins over the urea concentration range of 0 M to 2.6 M or 0.816 M, respectively. Induced change in the secondary structure is represented by change in CD signal at 220 or 204 nm. The data were normalized and plotted *versus* the relevant urea concentration, and the results indicated that the stabilization of the mutant holoflavodoxin is weaker than that of wild-type (Figure 2c). The UV-visible spectrum of the oxidized holoflavodoxin mutant is very similar to that of wild-type holoflavodoxin. However, a 2-nm blue shift of correlative characteristic peaks from 350 to 400 nm and a 4-nm red shift from 400 to 450 nm (Table 2) may have resulted from certain subtle changes in the hydrophobic environment with the substitution of the aromatic amino acid residues. 4,4′-Dianilino-1,1′-binaphthyl-5,5′-disulfonic acid (*bis*-ANS) is a fluorescence probe, and fluorescence intensity is enhanced by *bis*-ANS binding in the molten globular structure of proteins. As shown in Figure 2d, although both apoflavodoxin and holoflavodoxin mutants have hydrophobic clusters, the enhanced fluorescence emission of holoflavodoxin suggested that the latter has a more accessible hydrophobic core than the former. Figure 2e clearly shows the UV-visible spectrum of the fully reduced holoflavodoxin. However, no characteristic spectrum of the semiquinone form of the holoflavodoxin mutant was found during the anaerobic titration with sodium dithionite (Figure 2e). Thus, we suggested that a semiquinone form of the holoflavodoxin mutant is extremely unsteady compared with the wild-type holoflavodoxin.

### 2.4. Redox Potentials and Dissociation Constant of the Mutant Flavodoxin

**Figure 3:**
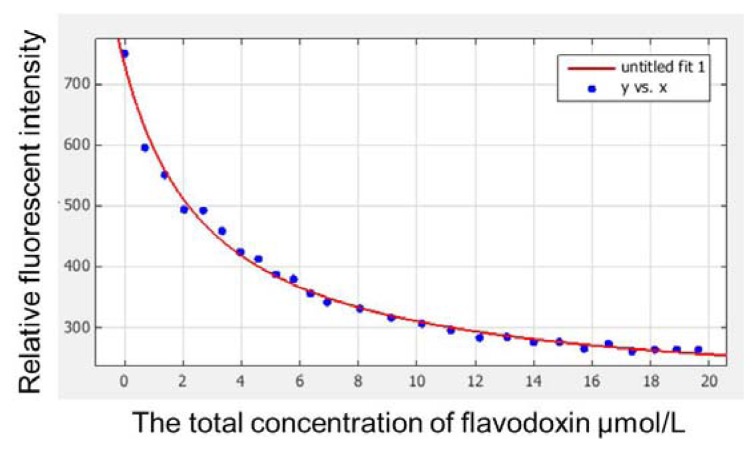
Fluorescent titration curve fitted by Matlab version 7.0. A 2.0 mL solution of 10 nM FMN in buffer B was titrated with 70 μM aliquots of apoflavodoxin in the dark. After equilibration for 3 min, the fluorescence emission at 530 nm upon excitation at 445 nm was recorded on a fluorescence spectrophotometer F4500 (Hitachi, Tokyo, Japan).

Redox potential is an important characteristic of a flavodoxin binding to a molecular non-covalent cofactor FMN. The oxidized FMN form is first reduced to semiquinone form when its isoalloxazin ring accepts an electron and a proton from a donor, and then hydroquinone is generated when one more electron is accepted [11]. No semiquinone flavodoxin component was observed during anaerobic titration, only the redox potentials of oxidized and fully reduced couple were detected. *E*_m,7_(fld) reached −360 mV, which is a larger negative value compared with that of wild protein (Table 2). *K*_d,ox_ reached 2.92 μM as determined by fitting fluorescent titration data (Figure 3), and *K*_d,hq_ was 43.604 mM by calculating the value of *K*_d,ox_ and *E*_m,7_(fld), which were both much higher than those of the wild-type flavodoxin, respectively (Table 2). The aromatic amino acids (W7, Y89, and W91) near the FMN-binding site in wild-type flavodoxin play a significant role in stabilizing the apoflavodoxin-FMN complex [12]. Thus, the weak binding of FMN and apoflavodoxin mutant should mainly arise from the lack of aromatic stack with the substitution of conserved aromatic amino acids to nonaromatic ones. The semiquinone form of the native flavodoxin is moderately stabilized with respect to the oxidized form, and the reduced form is strongly destabilized relative to both the oxidized and the semiquinone forms [10,12,13]. The steady semiquinone form of the native flavodoxin is associated with a complex hydrogen-bonded network formed between FMN_sq_ and apoflavodoxin [14,15]. However, the normal hydrogen-bonded network is broken with the substitution of “late amino acids”.

## 3. Experimental Section

Mutant and wild-type flavodoxin genes were synthesized according to the result of a rational design by Shanghai XuGuan Biotechnological Development Co., Ltd., Shanghai, China; 4,4′-dianilino-1,1′-binaphthyl-5,5′-disulfonic acid (*bis-*ANS) was obtained from Invitrogen; FMN (purity > 97%), benzyl viologen, 2-hydroxyethyl viologen, and methyl viologen were supplied by Tokyo Chemical Industry Ltd., Tokyo, Japan; and disulfonate, phenosafranine, safranine T, and sodium dithionite were purchased from China Pharmaceutical Group Shanghai Reagent Co., Ltd., Shanghai, China.

### 3.1. Expression and Purification of the Mutant Flavodoxin

Mutant and wild-type flavodoxin genes were subcloned into the *Nde* I and *Bam* HI site of pET28b (+) vector (Novagen, Darmstadt, Germany) using a standard protocol. The recombinant plasmid was sequenced and named pET28-bF, transformed into *E. coli* BL21 (DE3), and grown at 37 °C in Luria broth containing 100 μg/mL Kanamycin to OD_600_ (0.6). A solution of 1 mM isopropyl-d-thiogalactopyranoside (IPTG) was added, and the culture was grown for an additional 12 h. The cells were harvested by centrifugation at 4 °C (10,000× *g* for 5 min), washed three times with buffer A (50 mM NaH_2_PO_4_, 300 mM NaCl, 10 mM imidazole, pH 7.0), and frozen at −20 °C. Approximately 1 g of cell pellets was thawed at 4 °C, resuspended in 5 mL lysis buffer (buffer A containing 1 mg/mL lysozyme), and then lysed at 4 °C for 12 h. The lysate was sonicated at an ultrasonic power of 200 W on ice for 40 cycles (ultrasonic 10 s stop 10 s in each cycle), the homogenate was centrifuged at 4 °C (14,000× *g* for 30 min), and the supernatant was filtered and purified by affinity chromatography with Ni-NTA superflow cartridges (Qiagen, Hilden, Germany) using a standard protocol. The purity of the recombinant flavodoxin was confirmed by SDS-PAGE and Western blot assays.

### 3.2. Preparation of Apoflavodoxin and Holoflavodoxin

Apoflavodoxin was prepared by extraction of purified recombinant flavodoxin with 5% trichloroacetic acid and dissolution in buffer B (40 mM sodium phosphate, pH 7.0) [16]. Holoflavodoxin was reconstituted by incubating apoflavodoxin with 1.0 mM FMN, and dialysis was performed with buffer B at 4 °C for 24 h. The concentrations of apoflavodoxin and holoflavodoxin were determined by a standard bicinchonininc acid method.

### 3.3. Spectral Assay

The far-UV CD spectra of apoflavodoxin and holoflavodoxin were measured on a J810 CD instrument (Jasco, Tokyo, Japan) at 25 ± 1 °C. The UV-vis spectra of the oxidized form of the holoflavodoxin were recorded on a UV3310 spectrophotometer (Hitachi, Tokyo, Japan) at 25 ± 1 °C. The fluorescence spectra of 10 μM *bis*-ANS in the absence and presence of 10 μM mutant apoflavodoxin or holoflavodoxin were measured at 25 ± 1 °C on a FP 6500 spectrofluorometer (Jasco) with excitation at 390 nm. The absorbance properties of the semiquinone and fully reduced forms of holoflavodoxin were determined by anaerobic reduction in buffer B with sodium dithionite. Each sample contained within a sealable titration cuvette (Hellma, Mullheim, Germany) was made anaerobic by repeated cycles of vacuum evacuation and flushing with 99.999% nitrogen in an anaerobic glove box [15].

### 3.4. Determination of Redox Potentials and Dissociation Constant

The midpoint potential of the two-electron transfer of the mutant flavodoxin was determined spectrophotometrically at 25 ± 1 °C using equilibrated apoflavodoxin with redox indicator dyes in buffer B by anaerobic titration with sodium dithionite [16]. The indicator dyes utilized in the determination of the midpoint potential of ox/hq couple included indigo disulfonate (*E*_m,7_ = −116 mV), phenosafranine (*E*_m,7_ = −255 mV), safranine T (*E*_m,7_ = 276 mV), benzyl viologen (*E*_m,7_ = −359 mV), 2-hydroxyethyl viologen (*E*_m,7_ = −408 mV), and methyl viologen (*E*_m,7_ = −446 mV). Holoflavodoxin and dye concentrations varied from 2 to 70 μM. Concentrations of the various oxidized and reduced species in equilibrium during the anaerobic reduction were determined from the absorbance of the UV-vis spectra. The potential for the ox/hq couple of the flavodoxin was calculated from Equation (1) [17]:

(1) $$E_{\text{m},7}(\text{fld})=E_{\text{m},7}(\text{dye})+RT/nF\text{ln}[\text{oxidized dye}][\text{fully reduced fld}]/[\text{reduced dye}][\text{oxidized fld}]$$

where, *E*_m,7_(fld) is the midpoint potential of the oxidized and fully reduced couple of flavodoxin at pH 7.0, *E*_m,7_(dye) is the midpoint potential of the dye at pH 7.0, *R* is the gas constant, *T* is the absolute temperature, F is the faraday constant, and *n* is the number of electrochemical equivalents.

The dissociation constant for a fully oxidized state apoflavodoxin-FMN_ox_ complex (*K*_d,ox_) was determined by fluorescence spectroscopic titration method previously described by Bollen [18].

The dissociation constant was calculated by fitting the emission fluorescence data to Equation (2) [18]:

(2) $$F={dF}_{\text{end}}+F_{\delta }\{dC_{F}-1/2(C_{A}+K_{\text{d},\text{ox}}+{dC}_{F})+1/2{\left[{\left(C_{A}+K_{\text{d},\text{ox}}+{dC}_{F}\right)}^{2}-4C_{A}{dC}_{F}\right]}^{1/2}\}$$

where *F* is the observed fluorescence emission intensity after each addition, *F*_end_ is the remaining emission intensity at the end of the titration, *F_δ_* is the difference in emission intensity between 1 μM free FMN and 1 μM flavodoxin, *C_A_* is the total protein concentration after each addition, *K*_d,ox_ is the dissociation constant for apoflavodoxin-FMN_ox_ complex, *C_F_* is the starting concentration of FMN, and *d* is the dilution factor of this initial concentration (initial volume/total volume) after each addition. The dissociation constant for a fully reduced state apoflavodoxin-FMN_hq_ complex (*K*_d,hq_) and the free energy of the apoflavodoxin-FMN_ox_ complexes (Δ*G*_b,ox_) and apoflavodoxin-FMN_hq_ complexes (Δ*G*_b,hq_) can be calculated as described previously [10].

## 4. Conclusions

Natural evolution produces complex protein folds with a 20-amino acid alphabet. However, only a few amino acids are believed to have been involved in primitive protein synthesis [19]. Although a chorismate mutase composed entirely from a set of nine amino acids by directed evolution was created by Walter *et al.* [2], it does not belong to a primitive protein because of the presence of the late amino acid components of Met and Phe [2]. Thus, much effort should be contributed to recognize primitive proteins. Although conserved aromatic amino acids is important to the recognition and binding of apoflavodoxin-FMN complexes, multiple sequence alignment results showed that the most conserved site of native flavodoxins includes certain “early amino acids” [20]. In the present study, we constructed a flavodoxin mutant solely using “early amino acids” and evaluated its characteristic properties. Based on the results, a primitive protein was demonstrated to consist of “early amino acid” possessing cofactor-binding activity that can act as a two-electron transfer redox protein.

## Figures and Tables

**Table 1:** Substitute positions of the flavodoxin from *Megasphaera elsdenii* (PDBID: 2FZ5).

Amino acid position	6	7	16	36	54	57	70	71	86	89	91	96	97	100	120	127
Wild-type	Y	W	M	F	C	M	F	F	F	Y	W	W	M	W	M	C
Mutant	V	V	L	V	A	V	L	L	V	V	V	V	L	V	V	V
Secondary structure a	s	s	h	s	h	l	h	h	s	s	s	h	h	h	l	s

a s, h, and l represents β-sheet, α-helix, and loop, respectively.

**Table 2:** Comparison of the properties of wild-type and mutant-type flavodoxins.

	Absorption maxima (nm)	Redox potentials *E*_m,7_ (mV)	*K*_d,ox_ (μM)	Δ*G*_b,ox_ (kcal/mol)	*K*_d,hq_ (μM)	Δ*G*_b,hq_ (kcal/mol)
FMN	266, 373, 445 a	−207 a	–	–	–	–
Wild-type	272, 378, 446 b	−244 b	0.00047 b	−12.7 b	8.1 b	−11.0 b
Mutant-type	274, 376, 450	−360	2.92	−7.52	43,604	−1.85

a [8,9];

b [10].
